# Activation of ERK1/2 Causes Pazopanib Resistance via Downregulation of *DUSP6* in Synovial Sarcoma Cells

**DOI:** 10.1038/srep45332

**Published:** 2017-03-28

**Authors:** Nobuhiko Yokoyama, Tomoya Matsunobu, Yoshihiro Matsumoto, Jun-ichi Fukushi, Makoto Endo, Mihoko Hatano, Akira Nabeshima, Suguru Fukushima, Seiji Okada, Yukihide Iwamoto

**Affiliations:** 1 Department of Orthopaedic Surgery, Graduate School of Medical Sciences, Kyushu University, Japan; 2 Department of Advanced Medical Initiatives, Graduate School of Medical Sciences, Kyushu University, Japan.

## Abstract

Synovial sarcoma (SS) is a rare high-grade malignant mesenchymal tumour with a relatively poor prognosis despite intensive multimodal therapy. Although pazopanib, a multi-kinase inhibitor, is often used for advanced SS, most cases eventually become resistant to pazopanib. In the present study, we investigated the mechanisms of acquired pazopanib resistance in SS. To examine acquired pazopanib resistance, two SS cell lines, SYO-1 and HS-SY-II, were isolated after multiple selection steps with increasing concentrations of pazopanib. SYO-1 was also used *in vivo*. Then, pazopanib-resistant clones were investigated to assess potential mechanisms of acquired pazopanib resistance. Stable pazopanib-resistant clones were established and exhibited enhanced cell cycle progression, cell growth with increased ERK1/2 phosphorylation, and higher sensitivity than parental cells to a MEK-inhibitor, trametinib, both *in vitro* and *in vivo*. Furthermore, addition of low-dose trametinib partially reversed the pazopanib resistance. In the pazopanib-resistant clones, dual specificity phosphatase 6 (*DUSP6*) was downregulated. Inhibition of *DUSP6* expression in parental HS-SY-II cells partially recapitulated acquired pazopanib resistance. Acquired pazopanib resistance in SS was associated with activation of ERK1/2 through downregulation of *DUSP6* expression. Simultaneous treatment with pazopanib and a MEK inhibitor could be a promising strategy to overcome pazopanib resistance in SS.

Synovial sarcoma (SS) accounts for 7–10% of soft-tissue sarcomas and has a characteristic chromosomal translocation, t(X;18)(p11.2;q11.2), coding the chimeric protein SYT-SSX^1^. The treatment of SS consists of adjuvant chemotherapy, radical surgery, and/or radiotherapy. Several active agents against SS have been reported, and in general, doxorubicin- or ifosfamide-based regimens are applied as first-line adjuvant chemotherapies in SS^2^,^3^. Despite intensive treatment, patients with this tumour have relatively poor prognoses, with 5-year overall survival rates of 50–80%^4^,^5^,^6^.

Pazopanib is a multi-kinase inhibitor that inhibits several receptor tyrosine kinases (RTKs) such as platelet-derived growth factor receptor α (PDGFRα), PDGFRβ, vascular endothelial growth factor (VEGF) receptor 1 (VEGFR1), VEGFR2, VEGFR3, and c-Kit^7^,^8^. In the PALETTE study, a randomised, double-blinded, placebo-controlled phase III trial of pazopanib in pretreated metastatic sarcoma, pazopanib resulted in a statistically significant improvement in progression-free survival (PFS) of approximately 3 months^9^. Based on this result, pazopanib became a promising agent for second-line chemotherapy in patients with advanced soft-tissue sarcomas, including SS. However, disease progression eventually occurs in the majority of patients due to the development of pazopanib resistance. The mechanisms of acquired pazopanib resistance in SS have been minimally investigated and remain unclear, and it is important to elucidate the underlying mechanisms so as to define new therapeutic strategies for SS.

The present study was undertaken in order to establish pazopanib-resistant SS cells and examine the mechanisms of acquired pazopanib resistance.

## Results

### Establishment of pazopanib-resistant SS clones

**Figure 1:**
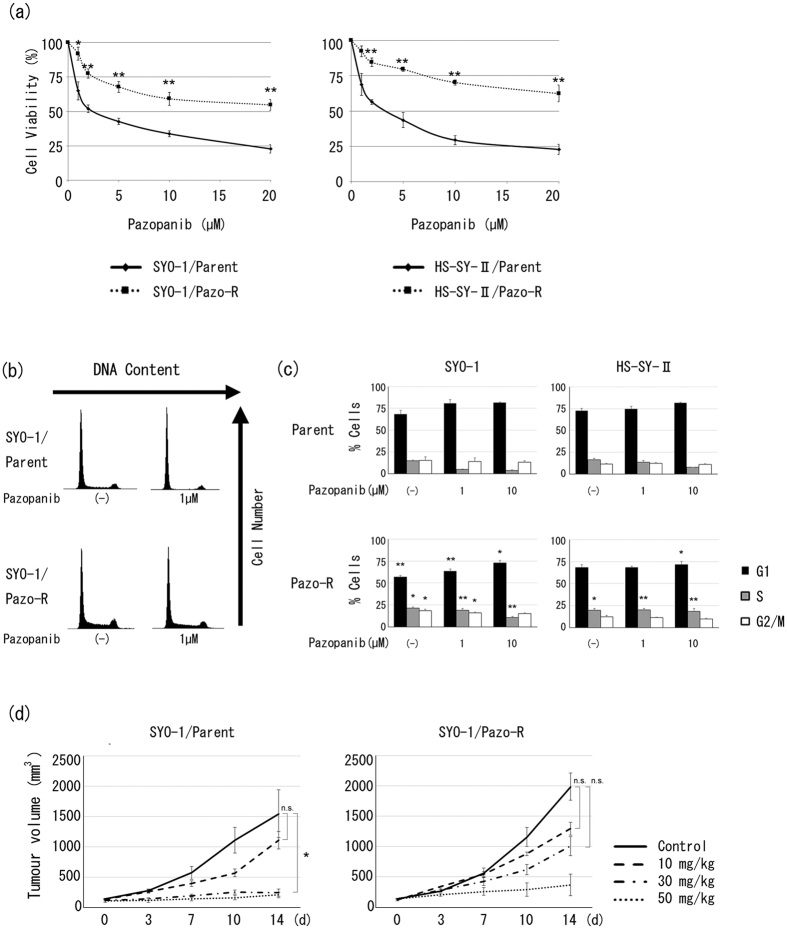
Establishment of pazopanib-resistant SS clones. (**a**) SS clones were incubated with various doses of pazopanib for 48 hours. Viable cells were counted using a Z1 Coulter particle counter. Values represent mean ± S.D. *P < 0.05; **P < 0.01, vs parental clones. (**b,c**) Cells were incubated with pazopanib (0, 1, and 10 μM) for 24 hours and fixed in 70% ethanol. After staining with PI, the DNA content of each phase (e.g., G1, S, or G2/M) was analysed by flow cytometry. Values represent mean ± S.D. *P < 0.05; **P < 0.01, vs parental clones. (**d**) Pazopanib resistance of SYO-1 parental and pazopanib-resistant clones *in vivo*. Values represent mean ± S.D. *P < 0.05; n.s. not significant, vs pazopanib-free condition. Pazo-R represents pazopanib-resistant clone.

Continuous stepwise selection of the two SS cell lines was undertaken with up to 20 μM pazopanib, and development of pazopanib resistance was periodically tested using a chemosensitivity assay. After approximately 6 months, stable, highly pazopanib-resistant clones were established in both cell lines as depicted in Fig. 1a. It has been reported that pazopanib mainly targets PDGFRα and induces G1/S arrest by inhibition of the PDGFRα–PI3K–Akt pathway in SS cells^10^. We therefore performed cell cycle analysis using flow cytometry in order to determine the cell cycle status of pazopanib-resistant and parental cells in the presence or absence of pazopanib. In the presence of pazopanib, parental clones exhibited a decrease in the proportion of cells in S phase in a dose-dependent manner. On the other hand, pazopanib-resistant clones exhibited an increase in the proportion of cells in S phase and a decrease of those in G1 phase, regardless of pazopanib, in comparison with parental clones (Fig 1b and c). Then, we examined pazopanib resistance in a mouse model using SYO-1 clones, since of the two cell lines only SYO-1 is tumourigenic. The pazopanib-resistant xenograft mice treated with 30 mg/kg pazopanib showed no significant decrease in tumour burden, while the parental xenograft model showed a significant decrease (Fig. 1d). These results suggest that acquired pazopanib resistance in SS cells causes promotion of cell cycle progression and that resistance is preserved in a preclinical mice xenograft model.

### Pazopanib-resistant clones showed enhanced cell growth and activated phosphorylation of ERK1/2

**Figure 2:**
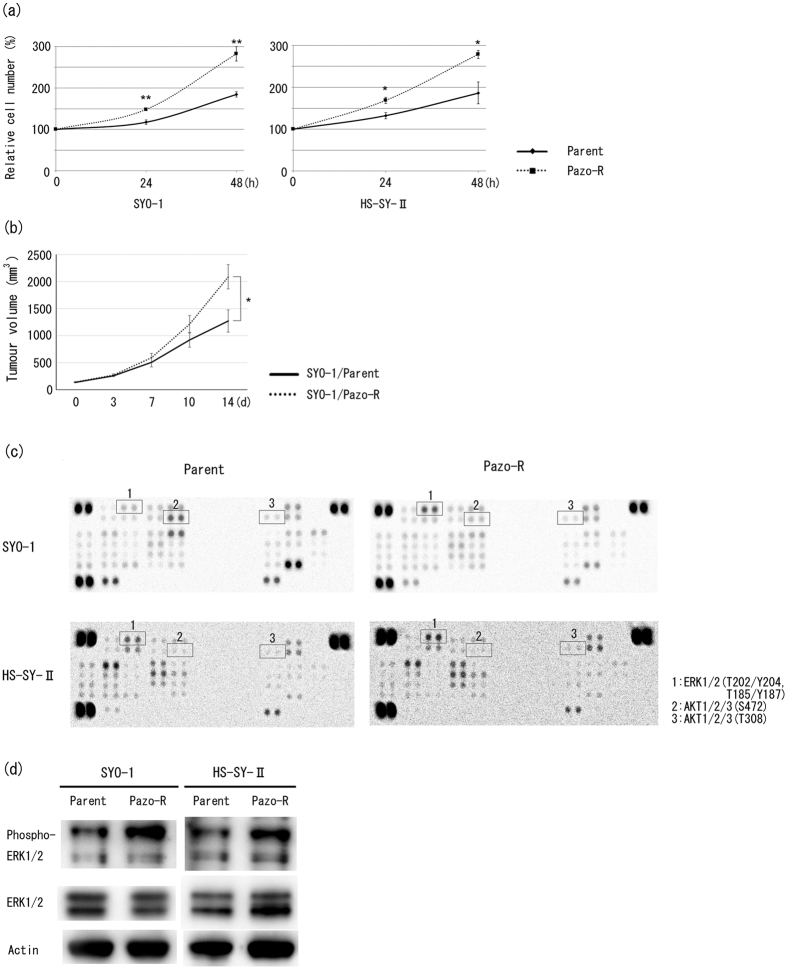
Phosphorylation profiles of cell signalling molecules in each clone. (**a**) SS clones were incubated for 48 hours. The relative number of viable cells was evaluated by CellTiter-Glo^TM^ Luminescent Cell Viability Assay. Values represent mean ± S.D. *P < 0.05; **P < 0.01, vs parental clones. (**b**) Tumourigenicity of SYO-1 parental and pazopanib-resistant clones. *P < 0.05, vs SYO-1 parental clone. (**c**) Multiplex analysis of the phosphorylation profile of the cell signalling molecules in SS clones. (**d**) Activated phosphorylation of ERK1/2 in pazopanib-resistant clones was confirmed by Western blot analysis using anti-ERK1/2 and anti-phospho-ERK1/2 antibodies. Pazo-R represents pazopanib-resistant clone.

We next performed a cell proliferation assay, since a previous study reported that accelerated cell cycle transitions were associated with deregulated cell proliferation^11^, and our cell cycle analysis showed that pazopanib-resistant clones were more frequently in S phase in the absence of pazopanib (Fig. 1b and c). Surprisingly, promotion of cell growth was observed in addition to cell cycle progression in pazopanib-resistant clones, compared with the parental clones in the absence of pazopanib (Fig. 2a). Consistent with *in vitro* experiments, mice inoculated with pazopanib-resistant SYO-1 cells showed a significant increase in tumour burden compared with the mice inoculated with parental cells (Fig. 2b). In order to address the mechanisms of cell cycle promotion and cell growth in pazopanib-resistant SS clones, as an initial approach we used a human phospho-antibody array (Human Phospho-Kinase Array, Proteome Profiler Array Kit) and semi-quantitate the levels of phosphorylation to study a subset of phosphorylation events in two SS cell lines to investigate signalling pathway profiles. In pazopanib-resistant clones, phosphorylation of ERK1/2 was increased in comparison with parental clones, while phosphorylation of Akt in the resistant clones was comparable or decreased (Fig. 2c and Table 1). Consistent with the kinase array results, Western blot analysis revealed increased ERK1/2 phosphorylation in pazopanib-resistant clones (Fig. 2d and Table 1).

### A MEK1/2 inhibitor strongly inhibited cell growth and partially reversed acquired pazopanib resistance in pazopanib-resistant clones

**Figure 3:**
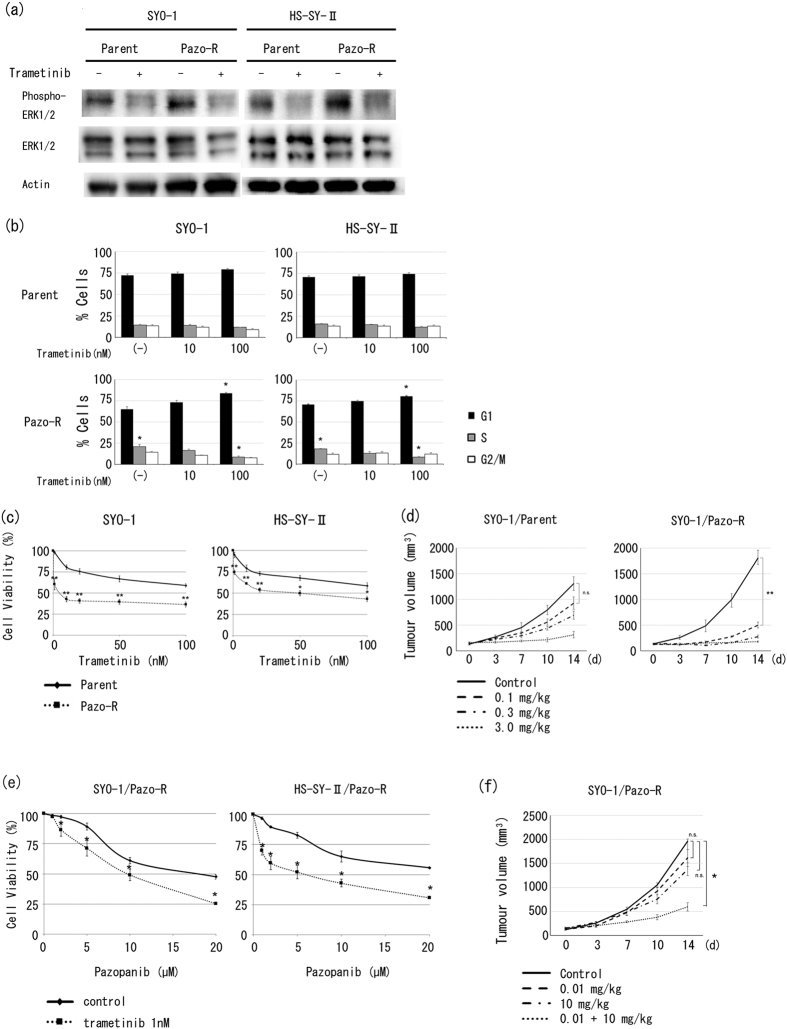
Trametinib, a MEK1/2 inhibitor, strongly inhibited growth and partially reversed pazopanib resistance of pazopanib-resistant clones. (**a**) Trametinib-induced inhibition of phosphorylation of ERK1/2 in SS clones was assessed by Western blot analysis with anti-ERK1/2 and anti-phospho-ERK1/2 antibodies. SS clones were pre-treated using 10 nM trametinib for 2 hours. (**b**) Cells were incubated with trametinib (10 nM and 100 nM) for 24 hours and fixed in 70% ethanol. After staining with PI, the DNA content of each fraction (e.g., G1, S, or G2/M) was analysed by flow cytometry. Values represent mean ± S.D. *P < 0.05; **P < 0.01, vs parental clones. (**c**) SS clones were incubated with various doses of trametinib for 48 hours. Viable cells were counted using a Z1 Coulter particle counter. Values represent mean ± S.D. *P < 0.05; **P < 0.01, vs parental clones. (**d**) Trametinib sensitivity of SYO-1 parental and pazopanib-resistant clones *in vivo*. **P < 0.01; n.s. not significant, vs trametinib-free condition. (**e**) Pazopanib-resistant SS clones were incubated with various doses of pazopanib and/or 1 nM trametinib for 48 hours. Viable cells were counted using a Z1 Coulter particle counter. Values represent mean ± S.D. *P < 0.05, vs trametinib-free condition. (**f**) Anti-tumour effect of 10 mg/kg pazopanib and/or 0.01 mg/kg trametinib in SYO-1 pazopanib-resistant clones *in vivo*. Values represent mean ± S.D. *P < 0.05, vs drug-free condition. Pazo-R represents pazopanib-resistant clone.

We examined whether activated ERK1/2 might be a good therapeutic target for pazopanib-resistant SS cells. To determine the role of ERK1/2 in SS cells, MAPK signalling pathways were inhibited with a chemical inhibitor, and cell cycle and cell proliferation were examined. We used trametinib, an oral, reversible, selective allosteric inhibitor of MEK1/2 activation and kinase activity, to inhibit the ERK signalling pathway^12^. First, Western blotting showed that phosphorylation of ERK1/2 in pazopanib-resistant clones was inhibited to the same extent by trametinib as parental clones (Fig. 3a), indicating that trametinib inhibited the ERK1/2 signalling pathway in both parental and pazopanib-resistant SS clones. Second, we examined the effect of trametinib on the cell cycle. Pazopanib-resistant clones showed a higher proportion of cells in the G1 phase than parental clones after treatment with 10 nM trametinib (Fig. 3b). Third, we performed a chemosensitivity assay with trametinib. Interestingly, trametinib more effectively inhibited the cell growth of pazopanib-resistant clones than parental clones (Fig. 3c). Fourth, we examined the *in vivo* efficacy of trametinib on pazopanib-resistant SYO-1 clones in the mouse model. As we expected, pazopanib-resistant xenograft mice treated with 0.1 mg/kg trametinib showed a significant decrease in tumour burden while parental xenograft mice treated with the same dose of trametinib showed no significant decrease (Fig. 3d). Finally, to examine whether inhibition of activated ERK1/2 would overcome pazopanib resistance, the pazopanib-resistant SS cells were simultaneously treated with pazopanib and a low dose of trametinib. In the pazopanib-resistant SS cells, even low-dose trametinib partially reversed the resistance to pazopanib chemosensitivity (Fig. 3e). In the SYO-1 mouse model, pazopanib-resistant clones showed higher sensitivity to 10 mg/kg pazopanib along with low-dose trametinib (Fig. 3f). These results suggest that trametinib can be an effective drug for pazopanib-resistant SS, and increased phosphorylation of ERK1/2 is a key to acquired pazopanib resistance.

### Mutational status of the RAS–RAF–MEK–ERK pathway and PDGFRα in pazopanib-resistant clones

**Figure 4:**
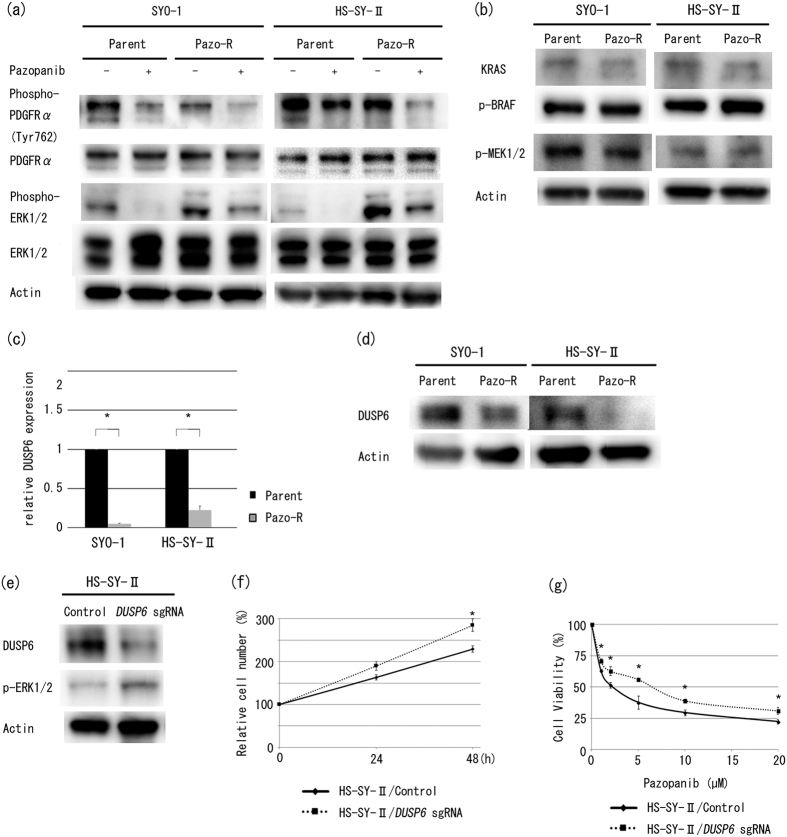
Mechanisms of increased phosphorylation of ERK1/2 in pazopanib-resistant SS cells. (**a**) Pazopanib-induced inhibition of phosphorylation of PDGFRα in SS clones was assessed by Western blot analysis with anti-PDGFRα, anti-phospho-PDGFRα, anti-ERK1/2, and anti-phospho ERK1/2 antibodies. SS clones were pre-treated with 5 μM pazopanib for 2 hours. (**b**) Phosphorylation of the RAS–RAF–MEK pathway in SS clones was assessed by Western blot analysis with anti-KRAS, anti-phospho-BRAF, and anti-phospho-MEK1/2 antibodies. (**c**) mRNA expression of *DUSP6* was assessed by quantitative RT-PCR. Values represent mean ± S.D. *P < 0.05, vs parental clones. (**d**) Protein expression of DUSP6 was assessed by Western blot analysis with anti-DUSP6 antibodies. (**e**) Protein expression of DUSP6 and phosphorylation of ERK1/2 in HS-SY-II control and CRISPR-treated cells were assessed by Western blot. (**f**) Control and CRISPR-treated HS-SY-II cells were incubated for 48 hours. The relative number of viable cells was evaluated by CellTiter-Glo^TM^ Luminescent Cell Viability Assay. (**g**) Control and CRISPR-treated HS-SY-II cells were incubated with various doses of pazopanib for 48 hours. Viable cells were counted using a Z1 Coulter particle counter. Values represent mean ± S.D. *P < 0.05; **P < 0.01, vs control clones. Pazo-R represents pazopanib-resistant clone, and *DUSP6* sgRNA represents CRISPR-treated cell.

To address the mechanism of activation of ERK1/2 in pazopanib-resistant SS cells, we examined aspects of the signalling pathway upstream of ERK1/2. First, we investigated the phosphorylation status of ERK1/2 and PDGFRα, the latter of which is a main target of pazopanib in SS since the PDGF signalling pathway can initiate the MAPK pathway and activate ERK1/2^13^. Interestingly, Western blot analysis showed that phosphorylation of PDGFRα was inhibited in all parental and pazopanib-resistant SS clones in the presence of pazopanib (Fig. 4a). However, pazopanib alone could not sufficiently inhibit phosphorylation of ERK1/2 in only pazopanib-resistant clones (Fig. 4a). Next, we examined kinases upstream of ERK1/2. The activation profile of the RAS–RAF–MEK–ERK pathway was similar between pazopanib-resistant and parental clones in Western blot analysis, with the exception of phospho-ERK1/2 (Fig. 4b). Furthermore, we conducted whole-exome sequencing in the pazopanib-resistant and parental clones to search for any activating mutations of the RAS–RAF–MEK–ERK pathway or any gatekeeper mutations of PDGFRα. There were no such mutations in either the pazopanib-resistant clones (Supplementary Figure 1) or parental clones (data not shown), and no common pazopanib-resistant-specific mutations (data not shown). These findings suggest that upstream signalling elements of ERK1/2, including gatekeeper mutations of PDGFRα, are not involved in the activation of ERK1/2 in pazopanib-resistant SS cells, and pazopanib-resistant clones are resistant to pazopanib inhibition of ERK1/2 phosphorylation. Next, we were interested in specific regulators of ERK1/2. It is known that sustained activation of ERK1/2 does not always correlate with upstream kinases, and dual specificity phosphatases (*DUSP*s) regulate MAPK activity^14^. We performed gene expression microarray analysis of SYO-1 parental and pazopanib-resistant clones to comprehensively evaluate molecules downstream from ERK1/2 in each clone. We identified downregulation of the *DUSP6* gene in the SYO-1 pazopanib-resistant sample using the same criteria described in the Methods section regarding all the *DUSP*s (Table 2). We next performed quantitative PCR and Western blot analysis to examine mRNA and protein expression of DUSP6 in all SS clones. The resistant clones showed lower expression levels of DUSP6 compared with the parental clones (Fig. 4c and d). Taken together, these findings suggest that activation of ERK1/2 is sustained, at least in part, via downregulation of *DUSP6* expression in pazopanib-resistant SS cells.

### Downregulation of *DUSP6* in parental SS cells recapitulates pazopanib resistance

To examine the relevance of *DUSP6* to ERK1/2 phosphorylation, cell growth promotion, and pazopanib resistance, we knocked down the expression of *DUSP6* in parental clones using the CRISPR/Cas9 system. As shown in Fig. 4e, phosphorylation of ERK1/2 was increased as a result of decreased *DUSP6* expression in HS-SY-II cells transfected with small guide RNA (sgRNA) targeting *DUSP6*.

We next examined the phenotypes of CRISPR-treated HS-SY-II cells. These cells showed enhanced cell growth (Fig. 4f), and in the chemosensitivity assay they exhibited a little greater resistance to pazopanib (Fig. 4g) than control cells. Taken together, these results suggest that downregulation of *DUSP6* caused enhanced cell growth and pazopanib resistance via activation of ERK1/2 in HS-SY-II.

## Discussion

Pazopanib is an oral multi-targeted tyrosine kinase inhibitor, and the first molecular target drug approved for soft-tissue sarcoma in Japan. Although a subgroup analysis showed that patients with synovial sarcoma, leiomyosarcoma, vascular tumours, alveolar soft part sarcoma, solitary fibrous tumour, and desmoplastic small round cell tumour had better progression-free survival with pazopanib^15^, most sarcomas eventually acquire drug resistance to pazopanib. Pazopanib targets VEGFRs, PDGFRs, and c-Kit^16^. Since previous studies found that immunohistochemistry of SS shows high protein expression of PDGFRs and VEGFRs^17^, pazopanib potentially offers advantages in the treatment of SS. Therefore, establishing cells with acquired pazopanib resistance and using SS cell lines to elucidate the mechanism underlying this drug resistance are critical to developing novel treatment strategies.

In the present study, the dose-sensitivity to pazopanib of parental SS clones was comparable to that in a previous study that showed an IC50 value between 2 and 10 μM (Fig. 1a)^10^. We succeeded in developing SS cell lines with acquired pazopanib resistance with an IC50 of more than 20 μM (Fig. 1a). It is reported that the recommended dose of pazopanib is 800 mg orally once daily, and a mean target trough concentration was 34 μM in patients with solid tumours^18^. Additionally, pazopanib of the almost same dose is administered in patients with renal cell carcinoma which is another adaption disease of pazopanib^19^. Therefore, we consider that our experiments at the concentration of 20 μM is clinically relevant.

Among the two SS cell lines utilised in this study, only SYO-1 is tumourigenic, and we therefore used it to perform preclinical animal experiments. The established pazopanib-resistant clones proved to have several characteristics. In addition to pazopanib resistance, promotion of cell growth was observed in pazopanib-resistant SS cell lines (Fig. 2a). Further, the resistant clones exhibited increases in phosphorylated ERK1/2 (Fig. 2c,d, and Table 1). In addition, we found that a couple of blots were also decreased commonly both SYO-1 and HS-SY-II resistant cells compared to their parental cells (Table 1). The semi-quantitation revealed that CREB, PRAS40, HSP60 and WNK1 were decreased prominently in SYO-1 (Table 1). Because CREB, PRAS40, HSP60 and WNK1 were considered to contribute to aggressive phenotypes such as tumour progression and metastases^20^,^21^,^22^,^23^, we speculated that their downregulation would be less likely to cause the aggressive phenotype of resistant clones. Further investigations are warranted to determine their roles in pazopanib resistance.

It is known that the MAPK pathway regulates diverse cellular processes such as cell growth, apoptosis, malignant transformation, and drug resistance^24^,^25^. MAPK signalling is tightly regulated and is activated primarily by extracellular growth factor stimulation. Activation of ERK1/2 can be induced by activation of its upstream kinases, RAS, RAF, and MEK1/2^26^. While many reports have shown that the MAPK pathway plays important roles in cancer drug resistance^25^, only a few have reported the involvement of the MAPK pathway in acquired tyrosine kinase inhibitor resistance^27^,^28^. The inactivation of the MAPK pathway has been proposed as one of the most promising approaches to overcoming acquired resistance to various cytotoxic drugs^29^,^30^. Indeed, we found that trametinib exerted greater antitumour effects against pazopanib-resistant SS cells than the parental cells, not only *in vitro* but also *in vivo* (Fig. 3b, c and d), and that low dose of trametinib along with pazopanib effectively inhibited the growth of pazopanib-resistant SS clones *in vitro* (Fig. 3e) and *in vivo* (Fig. 3f). Thus our data as well as those of others suggest that the MAPK pathway is a good molecular target in cases of acquired pazopanib resistance in SS. A phase II clinical trial has just been initiated to evaluate trametinib in combination with pazopanib in patients with advanced gastrointestinal stromal tumour (GIST) (ClinicalTrials.gov Identifier: NCT02342600). Similarly, simultaneous administration of trametinib and pazopanib was found to exhibit anti-tumour activity in a preclinical thyroid cancer model^31^. Further research regarding the clinical applications of trametinib in patients with advanced SS is required.

The causative molecular mechanisms of increased phosphorylated ERK1/2 have been proposed to be the activation of kinases upstream of ERK1/2^26^ and/or the inhibition of downstream phosphatases^32^. With regard to upstream molecules in the MAPK pathway, gatekeeper mutations of PDGFRα cause phosphorylation of ERK1/2 through the RAS–RAF–MEK pathway^13^. It has been reported that in cases of GIST, gatekeeper mutations of PDGFRα are frequently observed in resistance to other tyrosine kinase inhibitors such as imatinib^33^. However, a number of our findings suggest that it is unlikely that activation of ERK1/2 results from upstream signalling elements and activating mutations in acquired pazopanib-resistant SS cells: PDGFRα was not activated more in pazopanib-resistant SS cells than in parental SS cells (Fig. 4a), pazopanib was still able to inhibit the phosphorylation of PDGFRα in pazopanib-resistant SS cells (Fig. 4a), phosphorylation of ERK1/2 was partially inhibited by pazopanib (Fig. 4a), and there were no identified gatekeeper mutations of PDGFRα in both of the resistant clones (Supplementary Figure 1). Furthermore, Western blot analysis showed no differences between pazopanib-resistant and parental SS cells in expression levels and phosphorylation of KRAS, BRAF, and MEK1/2 (Fig. 4b).

With regard to downstream specific regulators in the MAPK pathway, DUSPs have been reported to modulate the duration, magnitude, and subcellular compartmentalization of MAPK activity^14^. It is known that DUSP dephosphorylates and inactivates MAP kinases^32^. Using a microarray system, we found that *DUSP6* was down-regulated in pazopanib-resistant SYO-1 cells (Table 2). Consistent with this microarray result, mRNA expression and protein expression of DUSP6 were down-regulated in all pazopanib-resistant clones (Fig. 4c and d). Forced knockdown of *DUSP6* in parental HS-SY-II cells reproduced the increases in ERK1/2 phosphorylation (Fig. 4e). A previous study showed that DUSP6 negatively and specifically modulated ERK1/2 kinase activity^34^. It has also been reported that down-regulation of *DUSP6* expression is involved in drug resistance in ovarian cancer^35^, and up-regulation of *DUSP6* mediated by p53 caused a cellular senescent phenotype^36^. Taken together with our results, these findings suggest that increased ERK1/2 phosphorylation in pazopanib-resistant SS cells is at least in part due to down-regulation of *DUSP6* expression.

As mentioned above, it has been reported that the activated MAPK pathway is associated with acquired cancer drug resistance^25^. Harada *et al*. reported that acquired resistance to sorafenib, a multi-kinase inhibitor, resulted from the continuous activation of MAPK pathway^27^. In this study, we revealed that trametinib inhibited cell growth of pazopanib-resistant SS cells more than the parental cells (Fig. 3c). In addition, inhibition of ERK1/2 by low-dose trametinib partially reversed pazopanib resistance *in vitro* (Fig. 3e) and *in vivo* (Fig. 3f). Conversely, *DUSP6* knockdown in parental HS-SY-II cells apparently induced pazopanib resistance, accompanied by an increase in phosphorylated ERK1/2 (Fig. 4e and g), which recapitulated the acquired resistance to pazopanib in SS cells. Therefore, we conclude that prolonged exposure to pazopanib induces downregulation of *DUSP6* by an unknown mechanism, and sustained phosphorylation of ERK1/2 promotes cell cycle progression and proliferation in SS cells.

Bridgeman *et al*. reported that the addition of trametinib could overcome acquired sunitinib resistance in renal cell carcinoma xenograft models^37^, which supports our conclusion that inhibition of ERK1/2 phosphorylation may be a key to overcome the acquired resistance to antiangiogenic tyrosine kinase inhibitors.

In this study, we found that pazopanib-resistant cells exhibited a marked increase in phosphorylated ERK1/2 (Fig. 2d), and the efficacy of trametinib on inhibiting the cell growth of pazopanib-resistant clones increased compared with the parental clones (Fig. 3c). Furthermore, down-regulation in *DUSP6* expression levels were shown in pazopanib-resistant cell lines (Fig. 4c and d). These results suggest that the increase in ERK1/2 phosphorylation and down-regulation in *DUSP6* expression are certainly keys to acquired pazopanib resistance, but the mechanisms of DUSP6 down-regulation are unknown. Further investigations are needed to determine what factors are responsible for the emergence of DUSP6 down-regulation.

In conclusion, this is the first report to address acquired pazopanib resistance in soft-tissue sarcoma. This study reveals a critical role of canonical MAPK signalling in the acquisition of pazopanib resistance through the down-regulation of *DUSP6* in SS cells. MEK1/2 inhibition with trametinib is a promising strategy to overcome pazopanib resistance in SS.

## Methods

### Cell lines

The human SS cell lines HS-SY-II and SYO-1 were used in this study. HS-SY-II was kindly provided by Dr. Sonobe (Department of Pathology, Kochi University, Nangoku)^38^ and SYO-1 by Dr. Kawai (Department of Musculoskeletal Oncology and Rehabilitation, National Cancer Center Hospital, Tokyo)^39^. Each cell line was maintained in RPMI 1640 (Invitrogen, Carlsbad, CA, USA), supplemented with 10% fetal bovine serum (HyClone Laboratories, Inc., Logan, UT, USA), 100 units/mL penicillin, and 100 μg/mL streptomycin. The cells were incubated at 37 °C in a humidified atmosphere of 5% CO_2_.

### Reagents

The primary antibodies are summarised in Supplementary Table S1.

Pazopanib was purchased from SYNkinase (San Diego, CA, USA) and trametinib was purchased from MedChem Express (Monmouth Junction, NJ, USA).

### Establishment of pazopanib-resistant SS clones

Pazopanib-resistant SS clones were isolated after multiple selection steps in the presence of increasing concentrations of pazopanib for approximately 6 months. Pazopanib concentrations were increased from 1 to 20 μM, because 20 μM was the highest concentration in which pazopanib could be solubilised in tissue culture media, and 20 μM is clinically achievable and lower than the pazopanib concentration of 40 μM found clinically in patients^40^.

### Chemosensitivity assay

For the chemosensitivity assay, 2 × 10^5^ cells were seeded in 6-well plates. After 24 hours incubation, various concentrations of pazopanib or trametinib were added to the media. After 48 hours incubation, the number of cells was counted with a Z1 Coulter particle counter (Beckman Coulter, Brea, CA, USA)^41^.

### Cell cycle analysis by flow cytometry

Cells were harvested with pazopanib or trametinib, incubated at 37 °C overnight, washed with PBS twice, and fixed with 70% ethanol. Then cells were centrifuged, washed with PBS twice, resuspended in PBS with 10 μg/mL RNase A and 50 μg/mL propidium iodide, and incubated for 30 minutes on ice. Alterations in cell distribution were analysed using a BD Accuri^TM^ C6 Flow Cytometer (BD Biosciences, San Jose, CA, USA). For each sample, 20,000 events were scored.

### Mouse tumour xenograft model

Female 5-week-old BALB/C nude mice were obtained from Charles River Japan (Fukuoka, Japan) and maintained in a “specific pathogen”–free environment throughout the experiment. Each SYO-1 clone (1 × 10^7^) was suspended in 200 μl BD Matrigel^TM^ Basement Membrane Matrix obtained from BD Biosciences (San Jose, CA, USA) and injected subcutaneously. When the tumour volume reached 100–200 mm^3^, mice were randomly divided into three groups, each consisting of at least four mice. Pazopanib or trametinib suspended in dimethyl sulfoxide (DMSO) and PBS were administered orally three times weekly at 0, 10, 30, or 50 mg/kg, and 0, 0.1, 0.3, or 3 mg/kg, respectively. Tumour volume was measured twice a week by calipers using the following equation: tumour volume (mm^3^) = (length × width^2^) ×0.5. At the end of the experiments, all the animals were euthanised. Experiments involving animals were performed in compliance with the guidelines established by the Animal Care and Use Committee of Kyushu University. The Institutional Review Board at Kyushu University approved the use of xenograft model for this study

### Cell proliferation assay

Cells were seeded in 96-well plates at a density of 2 × 10^3^ cells per well, and the number of viable cells in each well was measured using the CellTiter-Glo^TM^ Luminescent Cell Viability Assay (Promega, Madison, WI, USA).

### Phospho-kinase analysis

Proteome Profiler Human Phospho-Kinase Array Kits were purchased from R&D Systems Inc. (Minneapolis, MN, USA). Each clone grown in a 10-cm culture dish was incubated with drug-free media for 24 hours and the cell lysate was prepared and analysed as previously described^42^. Semi-quantitation was performed with ATTO CS Analyzer 3.0 (ATTO, Motoasakusa, Taito, Tokyo, Japan)

### Western blot analysis

Cells were incubated with 5 μM pazopanib or 10 nM trametinib for 2 hours, washed twice with ice-cold PBS, scraped, and centrifuged in microcentrifuge tubes. The cells were lysed using CelLytic M (Sigma-Aldrich, St Louis, MO, USA) with a protease and phosphatase inhibitor cocktail (Complete Mini, PhosSTOP; Roche Diagnostics, Mannheim, Germany). Western blot analysis was performed as described previously^43^.

### Target selection and sequencing

Exome sequencing was conducted for six DNA samples from each clone. Genomic DNA was extracted using QIAamp DNA Mini Kit (QIAGEN, Hilden, Germany), sheared into approximately 150–200 bp fragments, and used to make a library for multiplexed paired-end sequencing (Illumina, Tokyo, Japan). The constructed library was hybridised to biotinylated cRNA oligonucleotide baits from the SureSelect Human All Exon V6 Kit (Agilent Technologies, Santa Clara, CA, USA) for exome capture. Targeted sequences were purified by magnetic beads, amplified, and sequenced on an Illumina HiSeq2500 platform in a paired-end 100-bp configuration.

### Mapping and single-nucleotide variant/Indel calling

Adapter sequences were removed from reads by cutadapt (v1.2.1). After quality control, reads were mapped to the reference human genome (hg19) using BWA (ver.0.7.10). The mapping result was corrected using Picard (ver.1.73) for removing duplicates, and using GATK (ver.1.6–13) for local alignment and quality score recalibration. Single-nucleotide variant (SNV) and Indel calls were performed with multi-sample calling using GATK, and filtered to coordinates with Variant Quality Score Recalibration (VQSR) passed and variant call quality score ≥ 30. Somatic SNV calls were performed by comparing tumour and normal pairs using SomaticSniper (v1.0.2.3). Annotations of SNVs and Indels were based on dbSNP142, CCDS (NCBI, Nov 2014), RefSeq (UCSC Genome Browser, Nov 2014), Gencode (UCSC Genome Browser, ver. 19), and 1000Genomes (Nov 2014). Variants were further filtered according to the following predicted functions: frameshift, nonsense, read-through, missense, deletion, insertion, or insertion-deletion.

### Gene expression microarrays

The total RNA was isolated from each cell using TRIzol Reagent (Invitrogen) and purified using SV Total RNA Isolation System (Promega). RNA samples were quantified by an ND-1000 spectrophotometer (NanoDrop Technologies, Wilmington, DE, USA) and the quality was confirmed with an Experion System (Bio-Rad Laboratories, Hercules, CA, USA). The cRNA was amplified, labelled, and hybridised to a 60-K Agilent 60-mer oligomicroarray. All hybridised microarray slides were scanned by an Agilent scanner. Relative hybridization intensities and background hybridization values were calculated using Agilent Feature Extraction Software (9.5.1.1).

### Data analysis and filter criteria

Raw signal intensities and flags for each probe were calculated from hybridization intensities (gProcessedSignal) and spot information (gIsSaturated, etc.), according to the procedures recommended by Agilent. (Flag criteria on GeneSpring Software. Absent (A): ‘Feature is not positive and significant’ and ‘Feature is not above background’. Marginal (M): ‘Feature is not Uniform’, ‘Feature is Saturated’, and ‘Feature is a population outlier’. Present (P): others.) The raw signal intensities of two samples were log_2_-transformed and normalised by quantile algorithm with the ‘preprocessCore’ library package^44^ of Bioconductor software^45^. We selected probes with a ‘P’ flag call in both samples. To identify up- or down-regulated genes, we calculated Z-scores^46^ and ratios (non-log scaled fold-change) from the normalised signal intensities of each probe for comparison between SYO-1 parental samples and pazopanib-resistant samples. Then we established criteria for regulated genes: up-regulated genes, Z-score ≥ 2.0 and ratio ≥ 1.5-fold; down-regulated genes, Z-score ≤ −2.0 and ratio ≤ 0.66. A total of 1107 probes were obtained.

### RNA preparation and quantitative PCR

Total RNA from each clone was extracted using the RNeasy Lipid Tissue Mini kit (QIAGEN). Quantitative PCR was carried out using a LightCycler 1.5 as previously described (Perfect Real Time, Takara Bio, Otsu, Japan)^47^. The primers are summarised in Supplementary Table S2. Data were standardised using GAPDH as a housekeeping gene. A negative control was also prepared using distilled water instead of a DNA template. The assay was performed in triplicate and was repeated in at least three separate experiments. The expression of mRNA was calculated using LightCycler version 3.5 software (Roche Diagnostics).

### CRISPR/Cas9 system

The following were purchased from Thermo Fisher Scientific (Waltham, MA, USA): template DNA designed for gRNA, MEGAshortscript^TM^ Kit, MEGAclear^TM^ Kit, Lipofectamine^TM^ CRISPRMAX^TM^ Transfection Reagent, and GeneArt^TM^ Platinum Cas9 Nuclease. Transfection to the parental clones was carried out using Cas9 protein and lipid-mediated transfection according to the manufacturer’s instructions^48^.

### Statistical analysis

The Wilcoxon signed-rank test was used for two-group comparisons. *P* < 0.05 was considered to be statistically significant. Data in graphs are given as means ± standard deviation (S.D.). All statistical analyses were performed with the Statistical Analysis System (SAS) software package (JMP9, SAS Institute Inc., Cary, NC, USA).

## Additional Information

**How to cite this article**: Yokoyama, N. *et al*. Activation of ERK1/2 Causes Pazopanib Resistance via Downregulation of *DUSP6* in Synovial Sarcoma Cells. *Sci. Rep.* **7**, 45332; doi: 10.1038/srep45332 (2017).

**Publisher's note:** Springer Nature remains neutral with regard to jurisdictional claims in published maps and institutional affiliations.

## Supplementary Material

Supplementary Figure 1

Supplementary Table 1

Supplementary Table 2

## Figures and Tables

**Table 1:** Result of semi-quantitation of all tested phospho protein in phospho-kinase array.

Target	SYO-1	SYO-1 pazo	HS-SY-II	HS-SY-II pazo
p38	27185	50538	22286	20333
ERK1/2	100019	288321	89270	97567
JNK1/2/3	77395	77065	24433	22714
GSK 3a/b	143858	108616	24208	23893
p53 (S46)	167783	171765	44826	48486
EGFR	39309	48547	20694	18995
MSK1/2	50319	95009	62443	43471
AMPKa1	72491	50196	17451	19173
Akt1/2/3 (S473)	274974	89608	17563	16950
Akt1/2/3 (T308)	48944	37706	16570	21254
p53 (S15)	118583	124327	54242	68071
TOR	48586	56227	38804	24692
CREB	102784	75882	180734	99465
HSP27	31474	36865	17000	15359
AMPKa2	81370	86270	63793	66588
b-catenin	293000	127186	17943	16732
p70 S6 kinase (T389)	14568	9719	12116	14177
p53	89306	75529	17425	16322
c-Jun	107244	40943	17061	21136
Src	46052	53229	26714	18600
Lyn	50851	59028	49061	34769
Lck	27997	34987	16145	15046
STAT2	112232	134533	95794	85316
STAT 5a	58079	67275	39774	40098
p70 S6 kinase (T421/S424)	47915	34216	16237	16797
RSK1/2/3	60293	40917	18258	20572
eNOS	25031	17306	14288	16073
Fyn	43334	50826	39685	27175
Yes	53604	53433	34761	23466
Fgr	28021	26194	15085	14106
STAT6	76754	96172	46382	47204
STAT 5b	51351	81959	22535	21215
STAT3	33042	20225	18115	19723
p27	31729	12446	11176	13434
PLC-g1	29054	19782	13090	13218
Hck	94072	64611	39452	23454
Chk-2	61067	59698	35305	23094
FAK	90811	63448	26943	17407
PDGFRb	28910	33964	13902	15861
STAT5a/b	62719	81523	22467	21518
STAT3	53225	25804	11957	13286
WNK1	698519	160631	29746	24156
PYK2	17108	10415	13935	15843
PRAS40	371550	136339	24842	15820
HSP60	298441	180503	88998	64145

Pazo represents pazopanib-resistant clone.

**Table 2:** Result of gene expression microarray of SYO-1 parental and pazopanib-resistant clones about DUSPs.

Probe ID	Gene Symbol	SYO-1/Parent signal	SYO-1/Pazo-R signal	Z score	ratio
A_23_P139704	DUSP6	4520.012333	1190.453333	−3.46108	0.263374
A_24_P182494	DUSP10	19.96949667	3.618179143	−1.4046	0.181185
A_23_P150018	DUSP5	207.195	93.61953667	−1.37885	0.451843
A_24_P367602	DUSP5P1	790.0663333	446.2433667	−1.32193	0.564818
A_23_P120254	DUSP22	906.2421	697.3351333	−0.60877	0.76948
A_23_P7896	DUSP22	26.76996667	18.45077333	−0.4864	0.689234
A_23_P376759	DUSP11	265.9668667	232.3668667	−0.24048	0.873668
A_23_P154771	DUSP15	922.3841	837.5061333	−0.22705	0.90798
A_33_P3331345	DUSP11	75.97520333	69.89328	−0.11843	0.919949
A_33_P3383656	DUSP28	678.1746333	653.9184667	−0.08842	0.964233
A_23_P90933	DUSP19	23.91798333	23.26554333	−0.04734	0.972722
A_23_P134935	DUSP4	250.8295	245.6786	−0.04314	0.979465
**Probe ID**	**Gene Symbol**	**SYO-1/Parent signal**	**SYO-1/Pazo-R signal**	**Z score**	**ratio**
A_24_P417189	DUSP9	66.95873	137.8416667	1.239243	2.058606
A_23_P143650	DUSP18	62.21632333	123.5135	1.176579	1.985227
A_33_P3272698	DUSP23	10.63196867	28.16985667	1.030808	2.649543
A_23_P110712	DUSP1	185.0368694	303.3611493	0.846196	1.639463
A_24_P189739	DUSP16	93.94441667	120.9678	0.429173	1.287653
A_33_P3359012	DUSP8	556.4892	669.6873667	0.422641	1.203415
A_24_P37409	DUSP2	111.2015	132.5071	0.295323	1.191595
A_23_P51508	DUSP12	2528.705333	2771.286333	0.233366	1.095931
A_32_P98298	DUSP8	21.00351333	21.97329333	0.187192	1.046172
A_23_P129956	DUSP3	1343.944667	1413.815333	0.127332	1.051989
A_23_P207537	DUSP14	1199.725	1237.316333	0.075948	1.031333
**Probe ID**	**Gene Symbol**	**SYO-1/Parent signal**	**SYO-1/Pazo-R signal**	**Z score**	**ratio**
A_23_P104471	DUSP13	2.957820333	2.977214667	NULL	NULL
A_23_P369090	DUSP15	2.988015333	3.645668667	NULL	NULL
A_23_P256077	DUSP21	3.389116667	3.486214667	NULL	NULL
A_23_P146134	DUSP26	9.328484	3.483556	NULL	NULL
A_33_P3294645	DUSP27	4.514359333	3.326208667	NULL	NULL
A_24_P51855	DUSP7	3.698769333	3.489807667	NULL	NULL
A_33_P3263625	DUSP8	14.8356	5.108212667	NULL	NULL

Pazo-R represents pazopanib-resistant clone.

## References

[b1] Fisher C. Synovial sarcoma. Ann Diagn Pathol 2, 401–421 (1998). 9930576 10.1016/s1092-9134(98)80042-7

[b2] Sleijfer S., Seynaeve C. & Verweij J. Using single-agent therapy in adult patients with advanced soft tissue sarcoma can still be considered standard care. The oncologist 10, 833–841 (2005). 16314294 10.1634/theoncologist.10-10-833

[b3] Tascilar M., Loos W. J., Seynaeve C., Verweij J. & Sleijfer S. The pharmacologic basis of ifosfamide use in adult patients with advanced soft tissue sarcomas. The oncologist 12, 1351–1360 (2007). 18055856 10.1634/theoncologist.12-11-1351

[b4] Sultan I. . Comparing children and adults with synovial sarcoma in the Surveillance, Epidemiology, and End Results program, 1983 to 2005: an analysis of 1268 patients. Cancer 115, 3537–3547 (2009). 19514087 10.1002/cncr.24424

[b5] Bergh P. . Synovial sarcoma: identification of low and high risk groups. Cancer 85, 2596–2607 (1999). 10375108 10.1002/(sici)1097-0142(19990615)85:12<2596::aid-cncr16>3.0.co;2-k

[b6] Spillane A. J., A’Hern R., Judson I. R., Fisher C. & Thomas J. M. Synovial sarcoma: a clinicopathologic, staging, and prognostic assessment. Journal of clinical oncology 18, 3794–3803 (2000). 11078492 10.1200/JCO.2000.18.22.3794

[b7] Kumar R. . Pharmacokinetic-pharmacodynamic correlation from mouse to human with pazopanib, a multikinase angiogenesis inhibitor with potent antitumor and antiangiogenic activity. Molecular cancer therapeutics 6, 2012–2021 (2007). 17620431 10.1158/1535-7163.MCT-07-0193

[b8] Sleijfer S. . Pazopanib, a multikinase angiogenesis inhibitor, in patients with relapsed or refractory advanced soft tissue sarcoma: a phase II study from the European organisation for research and treatment of cancer-soft tissue and bone sarcoma group (EORTC study 62043). Journal of clinical oncology 27, 3126–3132 (2009). 19451427 10.1200/JCO.2008.21.3223

[b9] van der Graaf W. T. . Pazopanib for metastatic soft-tissue sarcoma (PALETTE): a randomised, double-blind, placebo-controlled phase 3 trial. Lancet 379, 1879–1886 (2012). 22595799 10.1016/S0140-6736(12)60651-5

[b10] Hosaka S. . A novel multi-kinase inhibitor pazopanib suppresses growth of synovial sarcoma cells through inhibition of the PI3K-AKT pathway. Journal of orthopaedic research 30, 1493–1498 (2012). 22359392 10.1002/jor.22091

[b11] Evan G. I. & Vousden K. H. Proliferation, cell cycle and apoptosis in cancer. Nature 411, 342–348 (2001). 11357141 10.1038/35077213

[b12] Zeiser R. Trametinib. Recent results in cancer research. Fortschritte der Krebsforschung. Progres dans les recherches sur le cancer 201, 241–248 (2014). 24756797 10.1007/978-3-642-54490-3_15

[b13] Cartel N. J., Liu J., Wang J. & Post M. PDGF-BB-mediated activation of p42(MAPK) is independent of PDGF beta-receptor tyrosine phosphorylation. American journal of physiology. Lung cellular and molecular physiology 281, L786–798 (2001). 11557582 10.1152/ajplung.2001.281.4.L786

[b14] Camps M., Nichols A. & Arkinstall S. Dual specificity phosphatases: a gene family for control of MAP kinase function. FASEB J 14, 6–16 (2000). 10627275

[b15] Kasper B. . Long-term responders and survivors on pazopanib for advanced soft tissue sarcomas: subanalysis of two European Organisation for Research and Treatment of Cancer (EORTC) clinical trials 62043 and 62072. Annals of oncology 25, 719–724 (2014). 24504442 10.1093/annonc/mdt586 PMC4433518

[b16] Schutz F. A., Choueiri T. K. & Sternberg C. N. Pazopanib: Clinical development of a potent anti-angiogenic drug. Critical reviews in oncology/hematology 77, 163–171 (2011). 20456972 10.1016/j.critrevonc.2010.02.012

[b17] Kampmann E. . VEGFR2 predicts decreased patients survival in soft tissue sarcomas. Pathology, research and practice 211, 726–730 (2015). 10.1016/j.prp.2015.04.015 26298629

[b18] Hurwitz H. I. . Phase I trial of pazopanib in patients with advanced cancer. Clinical cancer research 15, 4220–4227 (2009). 19509175 10.1158/1078-0432.CCR-08-2740

[b19] Sternberg C. N. . Pazopanib in locally advanced or metastatic renal cell carcinoma: results of a randomized phase III trial. Journal of clinical oncology 28, 1061–1068 (2010). 20100962 10.1200/JCO.2009.23.9764

[b20] Xiao X., Li B. X., Mitton B., Ikeda A. & Sakamoto K. M. Targeting CREB for cancer therapy: friend or foe. Curr Cancer Drug Targets 10, 384–391 (2010). 20370681 10.2174/156800910791208535 PMC4206256

[b21] Moniz S. & Jordan P. Emerging roles for WNK kinases in cancer. Cell Mol Life Sci 67, 1265–1276 (2010). 20094755 10.1007/s00018-010-0261-6 PMC11115774

[b22] Lu Y. Z., Deng A. M., Li L. H., Liu G. Y. & Wu G. Y. Prognostic role of phospho-PRAS40 (Thr246) expression in gastric cancer. Archives of medical science 10, 149–153 (2014). 24701227 10.5114/aoms.2013.36927 PMC3953967

[b23] Li X. S., Xu Q., Fu X. Y. & Luo W. S. Heat shock protein 60 overexpression is associated with the progression and prognosis in gastric cancer. PloS one 9, e107507 (2014). 25207654 10.1371/journal.pone.0107507 PMC4160299

[b24] Boguslawski G., McGlynn P. W., Harvey K. A. & Kovala A. T. SU1498, an inhibitor of vascular endothelial growth factor receptor 2, causes accumulation of phosphorylated ERK kinases and inhibits their activity *in vivo* and *in vitro*. J Biol Chem 279, 5716–5724 (2004). 14625306 10.1074/jbc.M308625200

[b25] McCubrey J. A. . Roles of the Raf/MEK/ERK pathway in cell growth, malignant transformation and drug resistance. Biochim Biophys Acta 1773, 1263–1284 (2007). 17126425 10.1016/j.bbamcr.2006.10.001 PMC2696318

[b26] Wortzel I. & Seger R. The ERK Cascade: Distinct Functions within Various Subcellular Organelles. Genes Cancer 2, 195–209 (2011). 21779493 10.1177/1947601911407328 PMC3128630

[b27] Harada K., Miyake H., Kusuda Y. & Fujisawa M. Characterization of mechanism involved in acquired resistance to sorafenib in a mouse renal cell cancer RenCa model. Clinical & translational oncology 16, 801–806 (2014). 24356934 10.1007/s12094-013-1151-9

[b28] Isham C. R. . Development and characterization of a differentiated thyroid cancer cell line resistant to VEGFR-targeted kinase inhibitors. The Journal of clinical endocrinology and metabolism 99, E936–943 (2014). 24628546 10.1210/jc.2013-2658 PMC5393484

[b29] Alcala A. M. & Flaherty K. T. BRAF inhibitors for the treatment of metastatic melanoma: clinical trials and mechanisms of resistance. Clinical cancer research 18, 33–39 (2012). 22215904 10.1158/1078-0432.CCR-11-0997

[b30] Paillas S. . Targeting the p38 MAPK pathway inhibits irinotecan resistance in colon adenocarcinoma. Cancer research 71, 1041–1049 (2011). 21159664 10.1158/0008-5472.CAN-10-2726 PMC3304472

[b31] Ball D. W. . Trametinib with and without pazopanib has potent preclinical activity in thyroid cancer. Oncol Rep 34, 2319–2324 (2015). 26324075 10.3892/or.2015.4225 PMC4583528

[b32] Arkell R. S. . DUSP6/MKP-3 inactivates ERK1/2 but fails to bind and inactivate ERK5. Cell Signal 20, 836–843 (2008). 18280112 10.1016/j.cellsig.2007.12.014

[b33] Lee J. H., Kim Y., Choi J. W. & Kim Y. S. Correlation of imatinib resistance with the mutational status of KIT and PDGFRA genes in gastrointestinal stromal tumors: a meta-analysis. Journal of gastrointestinal and liver diseases 22, 413–418 (2013). 24369323

[b34] Camps M. . Catalytic activation of the phosphatase MKP-3 by ERK2 mitogen-activated protein kinase. Science 280, 1262–1265 (1998). 9596579 10.1126/science.280.5367.1262

[b35] Chan D. W. . Loss of MKP3 mediated by oxidative stress enhances tumorigenicity and chemoresistance of ovarian cancer cells. Carcinogenesis 29, 1742–1750 (2008). 18632752 10.1093/carcin/bgn167

[b36] Zhang H., Chi Y., Gao K., Zhang X. & Yao J. p53 protein-mediated up-regulation of MAP kinase phosphatase 3 (MKP-3) contributes to the establishment of the cellular senescent phenotype through dephosphorylation of extracellular signal-regulated kinase 1/2 (ERK1/2). J Biol Chem 290, 1129–1140 (2015). 25414256 10.1074/jbc.M114.590943 PMC4294480

[b37] Bridgeman V. L. . Preclinical Evidence That Trametinib Enhances the Response to Antiangiogenic Tyrosine Kinase Inhibitors in Renal Cell Carcinoma. Molecular cancer therapeutics 15, 172–183 (2016). 26487278 10.1158/1535-7163.MCT-15-0170

[b38] Sonobe H. . Establishment and characterization of a new human synovial sarcoma cell line, HS-SY-II. Laboratory investigation 67, 498–505 (1992). 1331610

[b39] Kawai A. . Establishment and characterization of a biphasic synovial sarcoma cell line, SYO-1. Cancer letters 204, 105–113 (2004). 14744540 10.1016/j.canlet.2003.09.031

[b40] Kumar R. & Suttle B. The importance of PK/PD data-key biological answers needed to evaluate the success of potential cancer therapeutics. Molecular cancer therapeutics 10, 2028 (2011). 22072811 10.1158/1535-7163.MCT-11-0728

[b41] Fujiwara-Okada Y. . Y-box binding protein-1 regulates cell proliferation and is associated with clinical outcomes of osteosarcoma. British journal of cancer 108, 836–847 (2013). 23462806 10.1038/bjc.2012.579 PMC3590655

[b42] Nabeshima A. . Tumour-associated macrophages correlate with poor prognosis in myxoid liposarcoma and promote cell motility and invasion via the HB-EGF-EGFR-PI3K/Akt pathways. British journal of cancer 112, 547–555 (2015). 25562433 10.1038/bjc.2014.637 PMC4453656

[b43] Hatano M. . Cadherin-11 regulates the metastasis of Ewing sarcoma cells to bone. Clin Exp Metastasis 32, 579–591 (2015). 26092671 10.1007/s10585-015-9729-y

[b44] Bolstad B. M., Irizarry R. A., Astrand M. & Speed T. P. A comparison of normalization methods for high density oligonucleotide array data based on variance and bias. Bioinformatics 19, 185–193 (2003). 12538238 10.1093/bioinformatics/19.2.185

[b45] Gentleman R. C. . Bioconductor: open software development for computational biology and bioinformatics. Genome Biol 5, R80 (2004). 15461798 10.1186/gb-2004-5-10-r80 PMC545600

[b46] Quackenbush J. Microarray data normalization and transformation. Nat Genet 32 Suppl, 496–501 (2002). 12454644 10.1038/ng1032

[b47] Fujiwara T. . Macrophage infiltration predicts a poor prognosis for human ewing sarcoma. Am J Pathol 179, 1157–1170 (2011). 21771572 10.1016/j.ajpath.2011.05.034 PMC3157220

[b48] Liang X. . Rapid and highly efficient mammalian cell engineering via Cas9 protein transfection. J Biotechnol 208, 44–53 (2015). 26003884 10.1016/j.jbiotec.2015.04.024

