# Supplementary material for: Activation of ERK1/2 Causes Pazopanib Resistance via Downregulation of DUSP6 in Synovial Sarcoma Cells
Source: Sci Rep. 2017 Mar 28;7:45332. doi: 10.1038/srep45332 (PMC5368598; doi:10.1038/srep45332)
Supplement: Supplementary Figure 1 [file srep45332-s1.doc]

**Activation of ERK1/2 Causes Pazopanib Resistance via Downregulation of *DUSP6* in Synovial Sarcoma Cells**

Nobuhiko Yokoyama^1^, Tomoya Matsunobu*^1^, Yoshihiro Matsumoto^1^, Jun-ichi Fukushi^1^, Makoto Endo^1^, Mihoko Hatano^1^, Akira Nabeshima^1^, Suguru Fukushima^1^, Seiji Okada^1,2^ and Yukihide Iwamoto^1^

Department of ^1^Orthopaedic Surgery, and ^2^Advanced Medical Initiatives, Graduate School of Medical Sciences, Kyushu University

**Running Title:** Pazopanib resistance in synovial sarcoma cells

***Correspondence:** Tomoya Matsunobu, Department of Orthopaedic Surgery, Graduate School of Medical Sciences, Kyushu University, 3-1-1 Maidashi, Higashi-ku, 812-8582 Fukuoka, Japan

Phone: 81-92-642-5488, Fax: 81-92-642-5507

E-mail: [matsunob@ortho.med.kyushu-u.ac.jp](mailto:matsunob@ortho.med.kyushu-u.ac.jp)

Figures: **4**, Tables: **2** Supplementary Figures: **1**, Tables: **2**

**Supplementary Figure . Mutational status of codon 842 of PDGFRα, hot spot of mutation.**

**
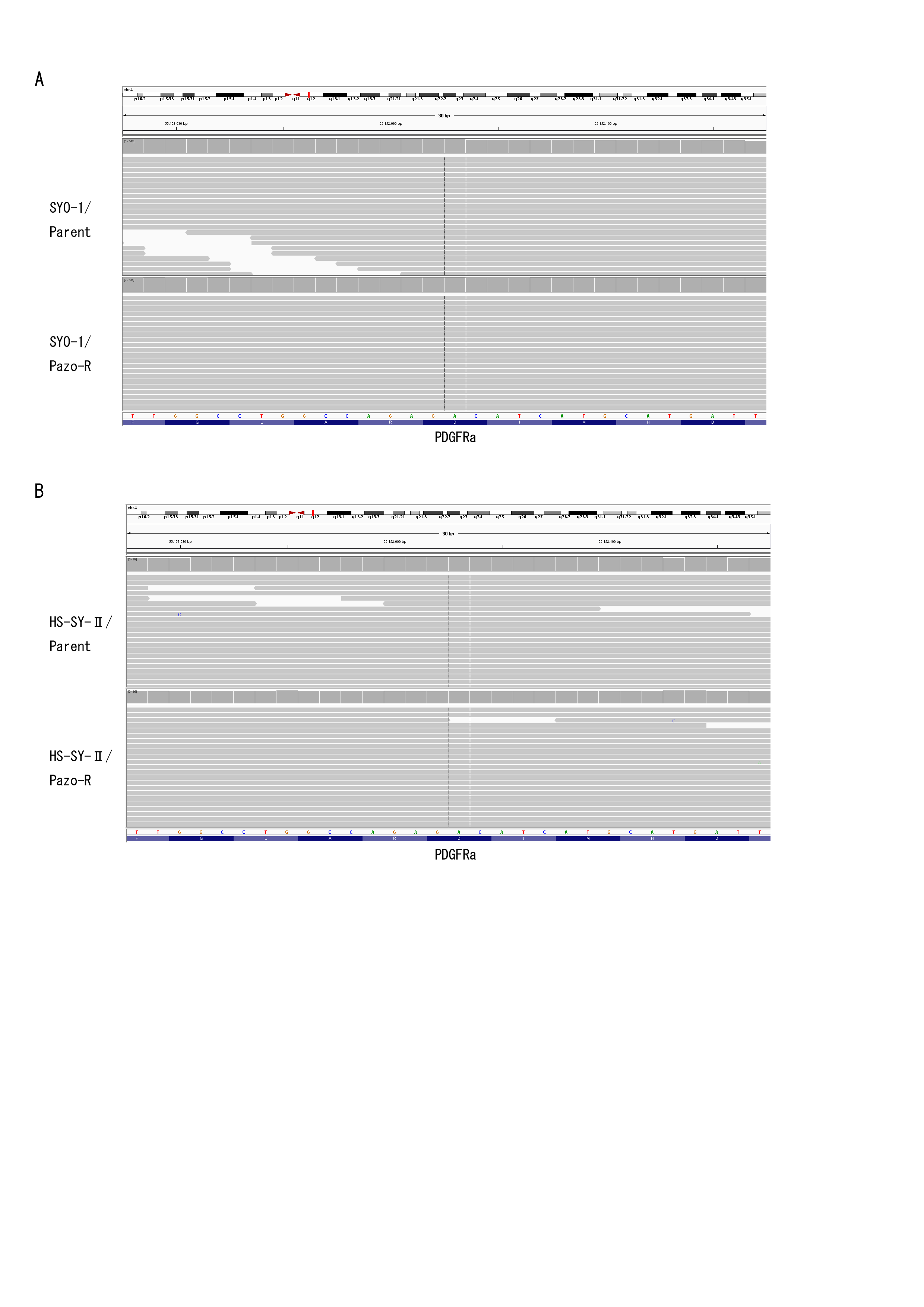
**

(A) Mutational status of SYO-1 parental and pazopanib-resistant clone.

(B) Mutational status of HS-SY-II parental and pazopanib-resistant clone.

Area surrounded by dot-line represents second base of codon 842 of PDGFRα.

Pazo-R represents pazopanib-resistant clone.
